# A Rare Case of Benign Metastasizing Leiomyoma Causing T11 Spinal Cord Compression: A Report and Literature Review

**DOI:** 10.7759/cureus.41875

**Published:** 2023-07-14

**Authors:** Gyuhee Seong, Simran Ahluwalia, Desiree Joy Anne Talabong, Burak Erdinc, Amena Mohiuddin, Maksim Agaronov, Edwin Chiu

**Affiliations:** 1 Department of Medicine, SUNY (State University of New York) Downstate Health Sciences University/Kings County Hospital, New York, USA; 2 School of Medicine, St. George's University, True Blue, GRD; 3 Department of Pathology, SUNY (State University of New York) Downstate Health Sciences University/Kings County Hospital, New York, USA; 4 Department of Hematology and Oncology, SUNY (State University of New York) Downstate Health Sciences University/Kings County Hospital, New York, USA

**Keywords:** leuprolide, cord compression, gnrh agonist, leiomyoma, extrauterine leiomyoma, benign metastasizing leiomyoma

## Abstract

Diagnosis of uterine smooth muscle tumors depends upon histologic characteristics as both benign and malignant share clinical features such as metastases. A benign metastasizing leiomyoma is a rare benign smooth muscle tumor that metastasizes to extrauterine sites with simultaneous uterine leiomyoma or previously biopsy-proven leiomyoma during myomectomy or hysterectomy. Benign metastasizing leiomyoma metastasizes outside the uterus, predominantly to the lungs and lymph nodes. However, the involvement of other organs, such as the heart, liver, spine, and soft tissue, is also reported. Here, we present a case of a 42-year-old woman with a history of uterine leiomyoma with prior myomectomy and hysterectomy, who presented with worsening back pain and lower extremity weakness and was found to have an acute cord compression, a serious complication caused by mass effect and a medical emergency that requires prompt attention to prevent permanent spinal cord damage. Sacral soft tissue biopsy and T11 spinal bone biopsy both demonstrated leiomyoma with immunostains positive for desmin, smooth muscle actin, and positive estrogen and progesterone receptors. No atypia, necrosis, and mitosis were identified. The patient had hepatic and pulmonary metastasis on imaging. The final diagnosis was benign metastasizing leiomyoma. There is no standard treatment for benign metastasizing leiomyoma. Both surgical and pharmacological approaches are employed. Although most cases are benign, there is a possibility for life-threatening complications. Benign metastasizing leiomyomas can be considered when multiple soft tissue tumors are found in premenopausal women with a history of uterine leiomyomas. Multidisciplinary discussion between oncologists, gynecologists, and relevant specialists is crucial in the optimal evaluation and treatment of benign metastasizing leiomyoma.

## Introduction

Uterine leiomyomas are one of the most common benign uterine tumors in women of reproductive age in the United States, and their prevalence is as high as 80% in certain ethnicities. Benign metastasizing leiomyoma occurs in predominately premenopausal women [1]. Only approximately 100 cases of benign metastasizing leiomyoma have been reported in the literature [2]. There are especially very few case reports of benign metastasizing leiomyoma causing spinal cord compression [3].

Here, we present a rare case of a 42-year-old female with a history of uterine leiomyoma with prior myomectomy and hysterectomy, who presented with back pain and lower extremity weakness, was found to have an acute cord compression, and diagnosed with benign metastasizing leiomyoma.

## Case presentation

A 42-year-old female from Jamaica with a past medical history significant for uterine leiomyoma, asthma, anemia, class III severe obesity with body mass index (BMI) of 45, and chronic sciatica, presented to the emergency department with back pain and bilateral lower extremity weakness. Past surgical history was notable for total abdominal myomectomy, hysterectomy, two cesarian sections, and partial gastrectomy. There was also a history of uterine and prostate cancer in the family. The patient reported ongoing back pain for the past year, the onset of which she attributes to lifting a heavy object. The patient recently noticed symptoms of sciatica. The patient was recently discharged from the hospital on methocarbamol (Robaxin) and a lidocaine patch for symptomatic treatment of her lower back pain but proceeded to experience difficulty walking with lower extremity numbness and tingling. There was also associated paresthesia in the perineal region, but the patient denied any urinary/bowel retention or incontinence.

**Figure 1:**
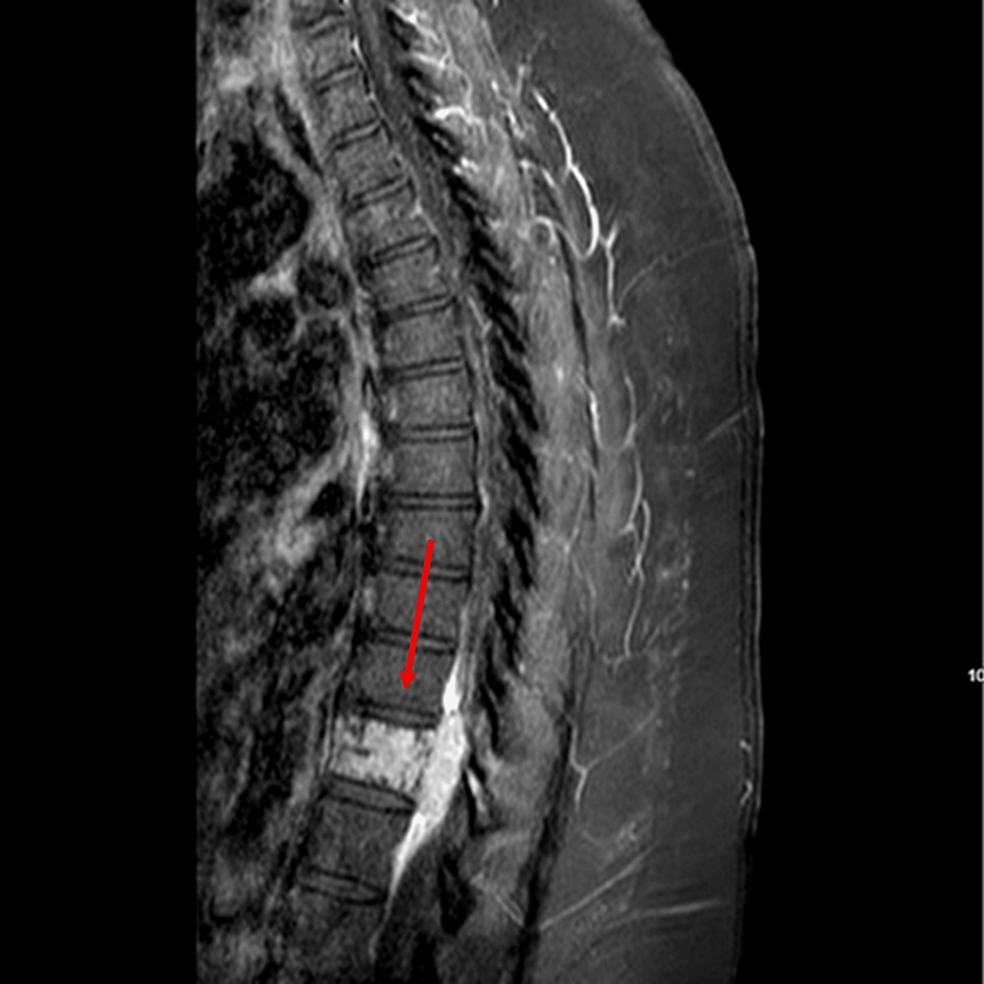
MRI thoracic spine with and without contrast Marked abnormality of the T11 vertebral body with diffuse enhancement and significant epidural disease in the spinal canal. Epidural soft tissue related to the T11 lesion (arrow) results in severe central canal stenosis and cord compression.

**Figure 2:**
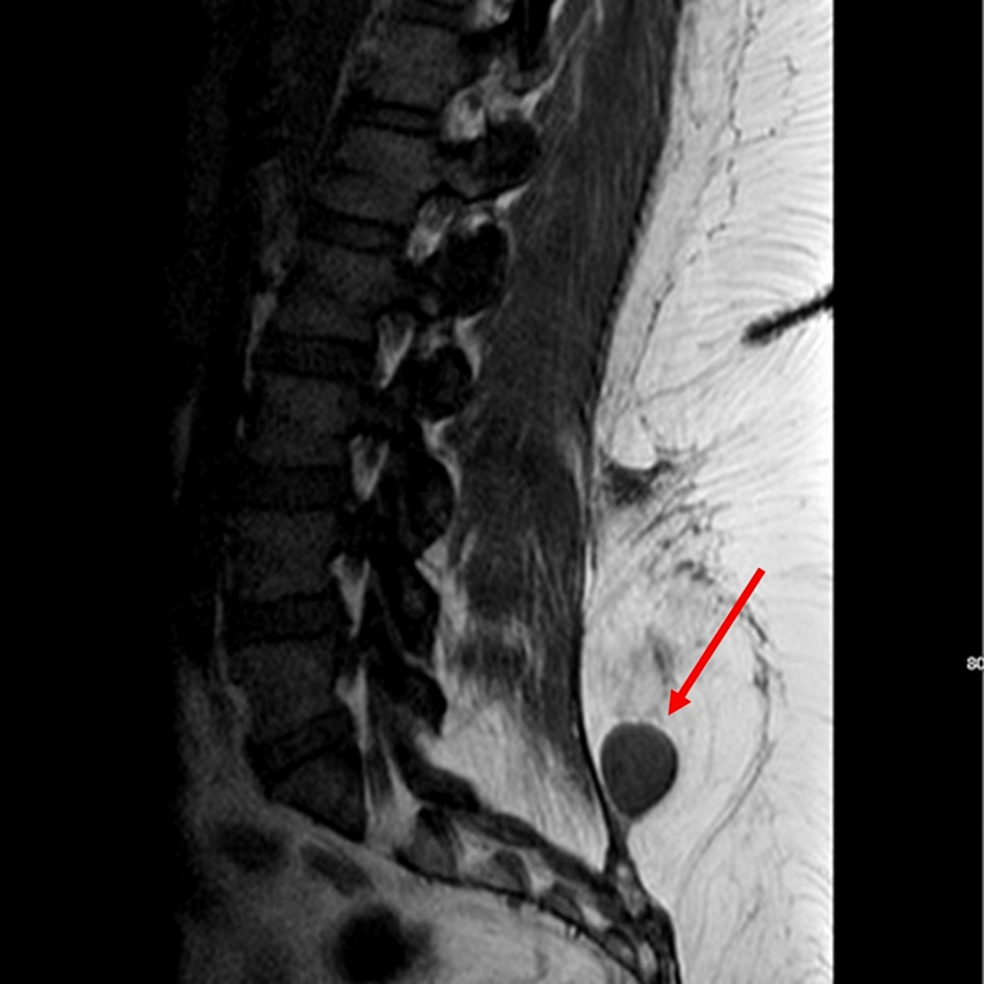
MRI lumbar spine with and without contrast Solid soft tissue nodule in the subcutaneous fat of the lower back at the level of the S1 segment (arrow). The lesion is superficial to the posterior lumbar fascia but demonstrates solid enhancement and is compatible with a neoplasm.

Vital signs and labs were unremarkable. Physical exam was notable for bilateral lower extremities weakness. Computed tomography (CT) abdomen/pelvis demonstrated a round soft tissue lesion measuring 2.6 x 2.4 x 2.8 cm adjacent to the inferior paraspinal muscles, T11 interosseous hemangioma involving the entire vertebral body. Magnetic resonance imaging (MRI) of the thoracic and lumbar spine confirmed abnormality of the T11 vertebral body with significant epidural extension into the spinal canal posterior to T11 and T12 vertebral bodies, with solid soft tissue nodule in subcutaneous fat of the lower back at S1 segment, superficial to the posterior lumbar fascia (Figures 1, 2).

**Figure 3:**
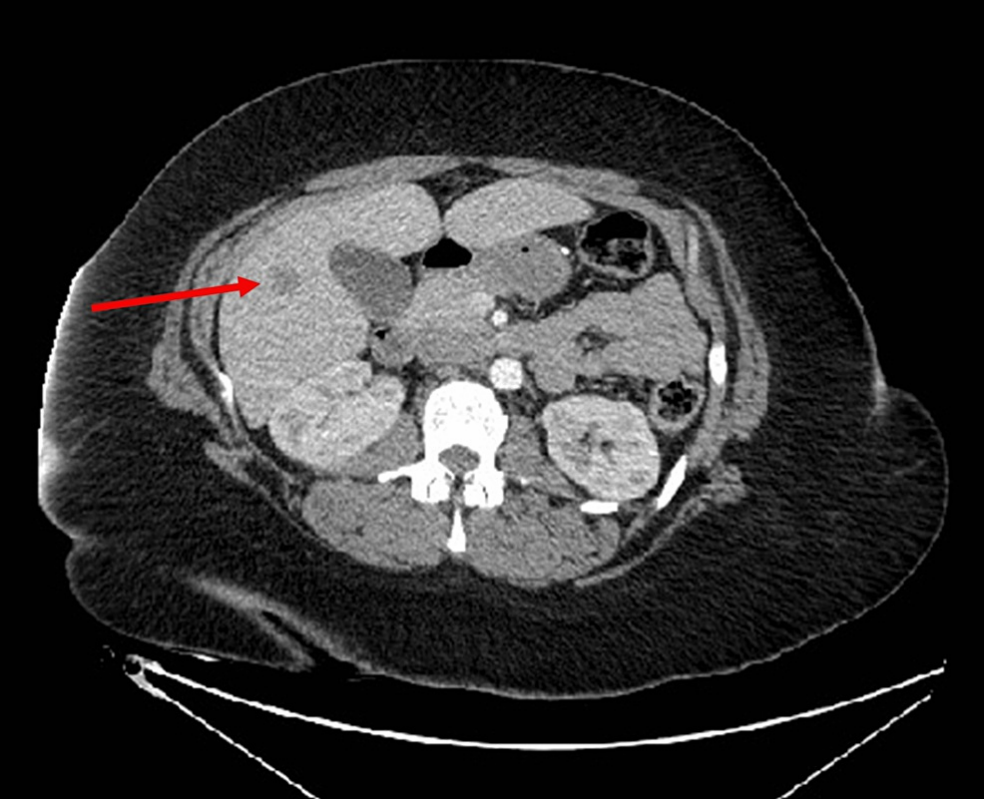
CT abdomen and pelvis with contrast A 1.5 cm hypodensity in segment 6 of the right lobe of the liver (arrow), which raises suspicion for metastatic disease in setting of malignancy.

**Figure 4:**
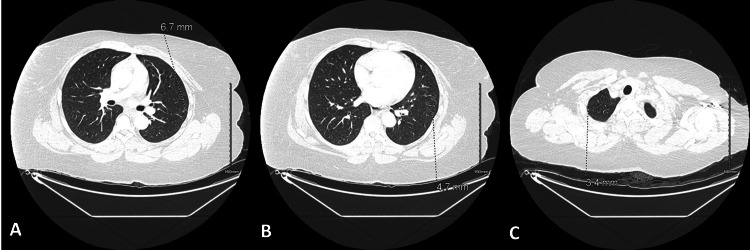
CT chest with contrast Scattered subcentimeter nodular densities in the lungs: (A) Left upper lobe 6.7 mm subsolid nodule; (B) Left lower lobe subsolid nodule measuring 4.7 mm; (C) 3.4 mm pleural-based nodule in the right lung apex.

CT abdomen/pelvis showed a hepatic lobe lesion measuring 1.5 x 1.4 x 1.5 cm (Figure 3). CT chest demonstrated scattered sub-centimeter nodular densities in the lungs, suggesting metastatic disease (Figure 4).

**Figure 5:**
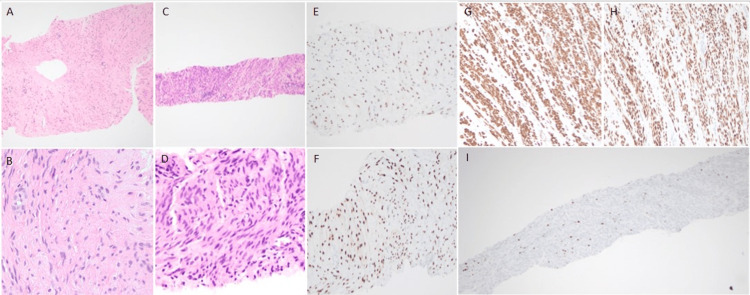
Biopsy of sacral soft tissue and T11 bone Histological examination of tissues with hematoxylin-eosin (H&E) staining under optical microscopy with 2x to 400x magnification was performed. Immunostaining for smooth muscle actin (SMA), desmin, vimentin, estrogen receptor (ER), progesterone receptor (PR), S100, SOX-10, melan A, and HMB45 was conducted on the formalin-fixed paraffin-embedded (FFPE) soft tissues. All antibodies for immunophenotyping are obtained from Ventana Medical Systems, Inc. (Oro Valley, Arizona, United States) and performed on Ventana XT instrument according to standard recommended protocols for each antibody. The biopsy of sacral soft tissue and T11 bone shows intersecting fascicles of monotonous benign spindle-shaped smooth muscle cells with distinct cellular borders, eosinophilic cytoplasm, and cigar-shaped nuclei with inconspicuous nucleoli. (A-D) No atypia, necrosis, and mitosis are identified in these biopsies. The biopsy from T11 appears more cellular. (C and D) Immunohistochemical stain shows tumor cells of the sacral soft tissue to be positive for smooth muscle markers such as vimentin, desmin, and SMA and both biopsies were positive for ER and PR. (E-H) T11 bone biopsy showed a low ki67 of 2-4%. (I) The spindle cell neoplasm from the sacral biopsy is negative for S100, SOX10, and melan A. T11 tumor biopsy showed similar immunohistochemical profile with negative HMB45.

Neurosurgery was consulted for cord compression and recommended to start dexamethasone for the management of cord compression. The patient was on an adequate pain management regimen throughout her hospital stay, including ibuprofen, tramadol, lidocaine patches, and Tylenol®. The patient was discharged to subacute rehabilitation and directed appropriately with referrals for Oncology, Neurosurgery, Neurology, and Gynecology. Medical oncology recommended a biopsy of the soft tissue mass in the sacrum, the result of which results returned after the patient was discharged. The soft tissue mass pathology in the sacrum showed spindle cell neoplasm with smooth muscle differentiation consistent with leiomyoma with immunostains positive for desmin, smooth muscle actin, and negative for Sox10, S100, and melan A. The vimentin stain was weakly positive (Figure 5).

History of uterine leiomyoma and histopathology results supported the diagnosis of benign metastasizing leiomyoma. Gonadotropin-releasing hormone (GnRH) agonist (leuprolide) was initiated to treat benign metastasizing leiomyoma. One and a half months later, the patient was hospitalized again after a fall with worsening back pain and paresthesia. The patient had another biopsy in T11 epidural bone to confirm the diagnosis of benign metastasizing leiomyoma.

Pathology of T11 bone biopsy showed spindle cell neoplasm consistent with leiomyoma. No necrosis or cytologic atypia was identified. Estrogen receptor (ER) was positive, progesterone receptor (PR) was positive, Ki-67: 2-4%, and HMB45 was negative. The immunohistochemical profile was similar to the prior biopsy from the sacrum (Figure 5).

Repeat CT of abdomen/pelvis with and without contrast showed a liver mass of 1.5 cm, stable from prior CT of abdomen/pelvis, and redemonstration of a nodule in the lower back soft tissues measuring 2.3 x 2 cm, decreased from prior 2.6 x 2.4 cm, representing a stable soft tissue mass.

The shrinkage of spinal lesion and improvement of pain and walking on GnRH agonist also supported the diagnosis of benign metastasizing leiomyoma.

## Discussion

Definition of benign metastasizing leiomyoma

Benign metastasizing leiomyoma is an uncommon condition, which Steiner initially reported in 1939. It is a cytologically and histologically benign smooth muscle tumor that metastasizes to extrauterine sites with simultaneous uterine leiomyoma or previously biopsy-proven leiomyoma during myomectomy or hysterectomy [4,5].

Uterine leiomyomas comprise 20-40% of benign uterine tumors in females of reproductive age in the United States. Benign metastasizing leiomyoma occurs in predominately premenopausal women [1]. Only approximately 100 cases have been reported in the literature [2]. There are three case reports of benign metastasizing leiomyoma causing spinal cord compression available on Pubmed [3,6,7].

They are reported to involve the lung, heart, lymph nodes, vascular channels, bone, spine, soft tissues, liver, bladder, skeletal muscles, esophagus, central nervous system, pelvic cavity, mediastinum, and retroperitoneum. Lungs and lymph nodes are one of the most affected sites of metastasis [1].

Presentation of benign metastasizing leiomyoma

Presentations of benign metastasizing leiomyoma can differ depending on the organs affected. Asymptomatic patients are frequently diagnosed incidentally by images or procedures performed for other indications. As in our case, patients with benign metastasizing leiomyoma in the spine experience leg pain and paresthesia, commonly in the posterior vertebral body [1].

Pathology of benign metastasizing leiomyoma

The WHO classification defines metastasizing leiomyoma as an extrauterine, well-demarcated nodular proliferation of benign-appearing smooth muscle that often occurs in patients with a history of prior hysterectomy or myomectomy [8]. The tumor cells are composed of benign, monotonous intersecting fascicles of smooth muscle cells that are cytologically described to have distinct cellular borders, eosinophilic cytoplasm, and oblong-shaped nuclei with inconspicuous nucleoli [8]. It may appear cellular or mixed with fat tissue [8]. The absence of atypia and necrosis with minimal to absent mitosis are other essential diagnostic criteria [1,8]. 

Uterine leiomyoma is characterized by whorls of benign smooth muscle cells. There are several subtypes of uterine leiomyoma, but the most common and usual subtype is defined by the absence of tumor necrosis, minimal mitosis, minimal cellular atypia, and invasion. The immunohistochemical profile of benign metastasizing leiomyoma is similar to uterine leiomyoma. They both have a low mitotic index, express smooth muscle markers such as vimentin, desmin, SMA, and muscle-specific actin, and are positive for ER and PR [8].

Of note, it would be crucial to differentiate benign metastasizing leiomyoma from malignancies such as leiomyosarcoma, which has an aggressive course [9]. The growth of the benign metastasizing leiomyoma is slow, and the size of the nodules is frequently reported to remain stable over a long period [1]. Notably, the absence of malignant features such as hypercellularity, high proliferative index, cytologic atypia, and necrosis defines histological features of benign metastasizing leiomyoma [10]. 

Differential diagnoses include solitary fibrous tumor (STAT6 positive, SMA negative, desmin negative) and schwannoma (S100 positive). Other spindle cell neoplasms such as melanoma, nerve sheath tumors, and sarcoma were ruled out using HMB45, melan-A, SOX10, and S100. Smooth muscle tumors of uncertain malignant potential (STUMP) need to be considered for differential diagnosis. STUMP refers to smooth tumors that exhibit histological characteristics exceeding diagnostic criteria for leiomyoma but insufficient to be diagnosed as leiomyosarcoma.

Pathogenesis of benign metastasizing leiomyoma

The exact pathogenesis of benign metastasizing leiomyoma is unknown. It is postulated that the cells of benign metastasizing leiomyoma originated from the uterus due to the strong relationship with uterine leiomyomas. Estrogens play a significant role in the origin of benign metastasizing leiomyoma as estrogen metabolites are promoting mutations. It has been hypothesized that benign metastasizing leiomyomas originated from a single site. A study by Patton et al. assessed clonality by analyzing the polymorphic CAG repeat sequence within the androgen receptors [11]. Their study supports the concept of clonal linkage between benign metastasizing leiomyoma and primary uterine leiomyoma. Benign metastasizing leiomyoma can also harbor the same mutation as its primary uterine leiomyoma [8,12]. Molecular studies were not performed in our case due to sample limitation because the blocks and slides of the previous myomectomy and hysterectomy done at an outside institution were not available for review and further testing.

Although benign metastasizing leiomyoma has been primarily associated with leiomyomas, other types of smooth muscle tumors in the uterus, such as leiomyosarcomas, endometrial stromal tumors, and smooth muscle tumors of uncertain malignant potential, are hypothesized to be related to benign metastasizing leiomyoma [1].

Treatment of benign metastasizing leiomyoma

Due to the lack of randomized clinical trials, there is no standard treatment to guide the optimal treatment of benign metastasizing leiomyoma. Asymptomatic patients with stable clinical courses might not require prompt treatment [1]. Both pharmacological and surgical approaches are utilized for benign metastasizing leiomyoma. GnRH agonist often decreases the tumor size. Surgical treatment includes hysterectomy or oophorectomy when a patient is diagnosed simultaneously with an intact uterus [1]. Each patient’s therapy is individualized, considering age, fertility, hormonal status, comorbidities, and symptoms [4].

Prognosis of benign metastasizing leiomyoma

Due to its benign nature, the prognosis of benign metastasizing leiomyoma is usually favorable [5]. However, recurrence and malignant degeneration in benign metastasizing leiomyoma are possible. Benign metastasizing leiomyoma in the spine has only a few reported cases, so the prognosis is yet uncertain.

## Conclusions

Benign metastasizing leiomyoma is a rare, cytologically and histologically benign, smooth muscle tumor that metastasizes to extrauterine sites with simultaneous uterine leiomyoma or previously biopsy-proven leiomyoma during myomectomy or hysterectomy. In this case, biopsies of soft tissue sacral mass and T11 bone showed benign histology yet the tumors had metastasized. Due to their location, they were compromising the patient's neurological function. The liver and lung nodules were incidental and currently stable. Although most cases of benign metastasizing leiomyoma are benign, life-threatening disease complications may occur. Our case presented an acute cord compression, a medical emergency that requires prompt attention to prevent permanent spinal cord damage. There is no standard treatment for benign metastasizing leiomyoma, but GnRH agonists often successfully shrink the tumor. In a premenopausal patient, metastatic lesions may not be pathognomonic for malignancy. A thorough histologic evaluation is essential. A multidisciplinary approach between oncologists, gynecologists, and relevant specialists in each case of benign metastasizing leiomyoma is crucial for optimal evaluation and treatment.
